# Human complement component C3 N-glycome changes in type 1 diabetes complications

**DOI:** 10.3389/fendo.2023.1101154

**Published:** 2023-05-24

**Authors:** Dinko Šoić, Jerko Štambuk, Marko Tijardović, Toma Keser, Gordan Lauc, Tomislav Bulum, Marijana Vučić Lovrenčić, Sandra Vučković Rebrina, Martina Tomić, Mislav Novokmet, Lea Smirčić-Duvnjak, Olga Gornik

**Affiliations:** ^1^ Faculty of Pharmacy and Biochemistry, University of Zagreb, Zagreb, Croatia; ^2^ Genos Glycoscience Research Laboratory, Zagreb, Croatia; ^3^ Department of Endocrinology, University Clinic Vuk Vrhovac, Zagreb, Croatia; ^4^ School of Medicine, University of Zagreb, Zagreb, Croatia; ^5^ Department of Medical Biochemistry and Laboratory Medicine, University Hospital Merkur, Zagreb, Croatia

**Keywords:** LC-MS, N-glycosylation, type 1 diabetes complications, complement component (C3), glycopeptides

## Abstract

**Aim:**

Changes in N-glycosylation have been described in numerous diseases and are being considered as biomarkers of ongoing pathological condition. Previous studies demonstrated the interrelation of N-glycosylation and type 1 diabetes (T1D), particularly linking serum N-glycan changes with complications accompanying the disease. Moreover, the role of complement component C3 in diabetic nephropathy and retinopathy has been implicated, and C3 N-glycome was found to be altered in young T1D patients. Therefore, we investigated associations between C3 N-glycan profiles and albuminuria and retinopathy accompanying T1D, as well as glycosylation connection with other known T1D complication risk factors.

**Research design and methods:**

Complement component C3 N-glycosylation profiles have been analyzed from 189 serum samples of T1D patients (median age 46) recruited at a Croatian hospital centre. Using our recently developed high-throughput method, relative abundances of all six of the C3 glycopeptides have been determined. Assessment of C3 N-glycome interconnection with T1D complications, hypertension, smoking status, estimated glomerular filtration rate (eGFR), glycaemic control and duration of the disease was done using linear modelling.

**Results:**

Significant changes of C3 N-glycome in severe albuminuria accompanying type 1 diabetes were observed, as well as in T1D subjects with hypertension. All except one of the C3 glycopeptides proved to be associated with measured HbA1c levels. One of the glycoforms was shown to be changed in non-proliferative T1D retinopathy. Smoking and eGFR showed no effect on C3 N-glycome. Furthermore, C3 N-glycosylation profile was shown to be independent of disease duration.

**Conclusion:**

This study empowered the role of C3 N-glycosylation in T1D, showing value in distinguishing subjects with different diabetic complications. Being independent of the disease duration, these changes may be associated with the disease onset, making C3 N-glycome a potential novel marker of the disease progression and severity.

## Introduction

Type 1 diabetes (T1D) related complications are divided into microvascular and macrovascular disorders, accounting for most of the morbidity and mortality associated with the disease (1). Microvascular complications of the disease manifest primarily as retinopathy, nephropathy and neuropathy. Diabetic nephropathy is the most common cause of renal failure in the western world, while diabetic retinopathy is the most common cause of acquired blindness. In addition, hypertension, smoking, glycaemic control and duration of the disease are all considered risk factors for complications accompanying T1D. Presence of microalbuminuria has also been shown to be highly predictive of progression to advanced stages of diabetic nephropathy (1–3). Hypertension is a common comorbidity in T1D with complex pathophysiology, and is also a well-recognized predictor of mortality and end-stage diabetic complications in this population (4). Active smoking was associated with increased risk for both incident hypertension and complications accompanying type 1 diabetes (1, 5). Among all the mentioned risk factors, poor glycaemic control (commonly measured by the level of nonenzymatic glycation of haemoglobin, HbA1c) is regarded as critical etiological factor in the development of the microvascular complications in T1D. Nevertheless, differences in HbA1c do not fully explain the variation of complications’ incidence and severity of the disease. Moreover, there is no HbA1c level below which complications are completely prevented, nor one above which complications are certain to develop (1, 2, 6). With prevalence of type 1 diabetes increasing, its long-term complications are more and more in the spotlight of. As early intervention improves outcome, new approaches in screening for T1D complications are of utmost importance (1–3).

Glycosylation is a ubiquitous co- and post-translational modification indirectly encoded by the genome and considerably influenced by environmental factors. It significantly contributes to structural heterogeneity of proteins and enriches its structure. The most common way of protein glycosylation is N-glycosylation which is characterized by the attachment of the glycan moity to the asparagine residue within the Asn-X-Ser/Thr protein motif. Changes in N-glycome profiles have been described in different diseases including type 1 diabetes and are being considered as biomarkers of ongoing pathological conditions (7–9). Our recent study revealed total plasma N-glycome changes in children with early onset type 1 diabetes, and one of the observed alterations was in the abundance of high-mannose N-glycans (10). Recent studies investigated serum N-glycosylation changes in adult type 1 diabetes patients with kidney disease and demonstrated that N-glycan profile of both total serum proteins and of IgG is altered. These studies showed that N-glycome is interrelated with estimated glomerular filtration rate (eGFR), as well as with HbA1c. Again, among the observed relations were high-mannose N-glycans (11, 12). Risk factors for diabetes complications such as smoking and hypertension also alter the N-glycome. It is known that smoking induces changes in glycosylation profiles of various glycoproteins, while IgG glycome is even suggested to change prior to the development of hypertension (13–15).

Due to the continual association of high-mannose glycans and T1D, and the fact that human complement component C3 is a protein occupied with these structures, our team recently studied C3 N-glycome in children and adolescents newly diagnosed with type 1 diabetes and found significant differences from their healthy siblings (10). C3 is central and most abundant protein of the complement system, a key system for immune surveillance with significant role in the pathogenesis of many diseases, including diabetes. Particularly, C3 contributes to the development of type 1 diabetes by enhancing the organ-specific autoimmune inflammatory processes (16, 17). In addition, plasma levels of C3 have been shown to be higher in patients with T1D than in healthy individuals (17, 18). Also, several studies have found that serum concentrations of mannose-binding lectin (MBL), a key activator of the lectin complement pathway, are significantly elevated in patients with type 1 diabetes (18–20).

There is also clear evidence for an involvement of complement in diabetic nephropathy and retinopathy. MBL levels were shown to be higher in T1D patients with albuminuria compared to normoalbuminuria patients (21). Type 1 diabetes patients with retinopathy also had higher serum MBL levels that those without the complication (22). In prospective studies, increased MBL levels even predicted microalbuminuria (23), as well as progression from macroalbuminuria to end stage renal disease (24). Furthermore, increased C3 concentrations in renal tissue seem to be in a relation with diabetic nephropathy, as glomerular deposition of activated C3 was observed in several animal studies (25, 26).

Despite all this evidence for involvement of complement component C3 and the glycosylation process in the pathogenesis of diabetes complications, C3 N-glycome has never been investigated in such population. Our study on young onset type 1 diabetes population was the first study of C3 N-glycosylation changes in any disease, and the results confirmed the relevance of C3 N-glycome analysis. Using our previously developed high-throughput glycoproteomic workflow for a site-specific N-glycosylation LC-MS analysis of human C3 (10), here we aimed to explore whether C3 N-glycan changes accompany T1D complications’ severity and progression. Additionally, since hypertension, smoking, HbA1c and duration of the disease are all considered major risk factors for T1D microvascular complications, we also inspected their effects on C3 N-glycome.

## Materials and methods

### Participants

All subjects participating in this study were recruited at Vuk Vrhovac University Clinic for Diabetes, Endocrinology and Metabolic Diseases of the Merkur University Hospital, Zagreb, Croatia. Cohort consisted of 189 blood serum samples. Study included type 1 diabetes subjects aged between 18 and 70 years (median age 46 years), on adequate treatment. Type 1 diabetes was defined as the onset of diabetes before age 35, positive diabetes associated autoantibodies and permanent insulin treatment initiated within 12 months after diagnosis.

Blood serum samples were collected in tubes for the purpose of N-glycan profiling. Biochemical tests such as high-sensitivity CRP, fasting serum glucose, renal function and lipid profile were performed and anthropometrical measurements collected. Blood pressure was measured twice in the sitting position with a mercury sphygmomanometer after a resting period of 10 minutes and expressed in mm Hg. Serum glucose was measured by enzymatic colorimetric method. Urinary albumin and haemoglobin A1c (HbA1c) were measured spectrophotometrically by turbidimetric immunoinhibition assays (AU680, Beckman-Coulter, USA and Tina-quant, Whole Blood, HbA1c Gen.2, Roche Diagnostics, USA, respectively). Results of HbA1c were IFCC-calibrated/NGSP-traceable and expressed in DCCT-equivalent (%). Data on serum standardized creatinine levels, age, sex and race were used to calculate eGFR using the Chronic Kidney Disease Epidemiology Collaboration (CKD-EPI) formula (27). Hypertension was defined as blood pressure at or above 140/90 mmHg, and/or the antihypertensive therapy. Subjects were assessed for common T1D complications (albuminuria and retinopathy) and divided into three categories per complication: without or with initial complications (1), moderate complications (2), and severe complications (3). Albuminuria categories were calculated based on urinary albumin excretion rate (AER), with criteria as follows: < 30 mg/24 h (1); 30 - 300 mg/24 h (2); >300 mg/24 h (3). Retinopathy status was assessed based on ocular examination findings, with categories as follows: no retinopathy (1); non-proliferative retinopathy (2); proliferative retinopathy (3). All subjects signed an informed consent and the ethical approvals from the local ethics committees were obtained. The study was conducted in accordance with the Declaration of Helsinki.

### C3 enrichment from human blood serum

C3 was isolated from the blood serum samples by Concanavalin A lectin affinity chromatography as described previously (10). In brief, C3 was enriched from 10 μl of serum in a high-throughput manner, using Concanavalin A-Sepharose 4B (Global Life Sciences Solutions, Marlborough, MA, United States) resin slurry placed into 96-well polypropylene filter (Orochem Technologies Inc., Naperville, IL, United States). The conditioned resin was loaded with serum samples that had been diluted 10-fold with binding buffer (20 mM TRIS pH 7.4, 0.5 M NaCl, 1 mM CaCl2, MnCl2), and the mixture was incubated while shaking overnight at 4°C. A vacuum manifold (Pall Corp., NY, USA) was used for washing of the lectin matrix. Afterwards, glycoproteins were eluted with elution buffer (200 mM methyl α-D-mannopyranoside (Sigma-Aldrich, St. Louis, MO, United States) in 0.1M acetic acid (Merck KgaA, Darmstadt, Germany), pH 3.0)) by low-speed centrifugation. The resulting eluates were then dried down immediately in a SpeedVac Vacuum Concentrator (Thermo Scientific, Waltham, MA, United States).

### Glycoprotein denaturation and Glu-C digestion

Dried enriched samples were subjected to denaturation by incubation with 2-propanol (Merck KgaA) in 0.1 M ammonium bicarbonate (15/85 v/v)(Acros Organics, Geel, Belgium) for 10 minutes at 60˚ C (28). To digest the samples, sequencing grade endoproteinase Glu-C (Sigma-Aldrich) was added in a ratio of 1:50 protease to protein (w/w), and the mixture was incubated for 18 hours at 37°C.

### HILIC-SPE glycopeptide enrichment

To enrich glycopeptides following Glu-C digestion, hydrophilic interaction liquid chromatography solid-phase extraction (HILIC-SPE) was used. A 50 mg/mL suspension of Chromabond HILIC silica beads (Macherey-Nagel GmbH & Co., Düren, Germany) was first prepared in 0.1% (v/v) TFA (Sigma-Aldrich) on a magnetic stirrer. Next, 100 μL of the suspension was added to each well of the Orochem OF1100 filter plate (Orochem Technologies Inc.). The wells were washed twice with 0.1% TFA (v/v) and then equilibrated with 0.1% TFA in 90% acetonitrile (ACN) (v/v) (VWR International, Radnor, PA, United States). The samples were diluted 10-fold with 0.1% TFA in ACN to a final concentration of 90% ACN (v/v) and applied to the preconditioned beads. After the removal of unbound impurities with 0.1% TFA in 90% ACN, enriched glycopeptides were eluted with 0.1% TFA into a PCR plate by low-speed centrifugation. The resulting eluates were immediately dried down in a SpeedVac Vacuum Concentrator (Thermo Scientific) and stored at −20°C until analysis.

### Nano-LC-ESI-MS analysis of purified glycopeptides

Analysis of glycopeptides was carried out on ACQUITY UPLC M-class nano-LC system (Waters, Milford, MA, United States) coupled to Compact Q-TOF mass spectrometer (Bruker Daltonik GmbH, Bremen, Germany) using CaptiveSpray ESI interface, and supported by nanoBooster dopant addition technology (Bruker Daltonik GmbH). After drying, glycopeptides were redissolved in 20 μL of ultrapure water and diluted 3-fold before loading onto an Acclaim PepMap C8 trap column (5 × 0.3 mm, 5 μm, 100 Å, Thermo-Scientific). Analytes were desalted for 3 min with 0.1% formic acid (FA) (v/v) at a 40 μL/min flow rate before being transferred to the analytical column. Prior to injecting each sample, trap column was washed with 20 μL of 25:75 isopropanol/ACN (v/v) followed by the same volume of 95% ACN (v/v). The glycopeptides were separated based on their peptide backbone using a SunShell C18 column (150 × 0.1 mm, 2.6 μm, ChromaNik Technologies Inc., Osaka, Japan) at a flow rate of 1 μL/min and a column temperature of 30˚C. The gradient began with 0% eluent B (0.1% FA in 80% ACN, v/v) and 100% eluent A (0.1% FA, v/v), increasing to 16% B within the first 3.5 minutes and remaining at 16% for another 1.5 minutes. Over the next 10 minutes, eluent B was further increased to 27%, and the gradient ended with a 6-minute wash using 100% eluent B, resulting in a total gradient time of 21 minutes. The column was subsequently equilibrated with 100% eluent A for 6 minutes.

The rolling average of spectra acquisition rate was set to 2 × 0.5 Hz, and the mass spectrometer operated in positive ion mode acquiring spectra in the m/z range of 600-2000. The capillary voltage was set at 1.5 kV, and the drying gas temperature was set to 150˚ C with a flow rate of 4 L/min. Nebulizing nitrogen gas was used at 0.2 bar and enhanced with ACN. The nano-LC-MS system was controlled by otofControl, MassLynx, and Compass HyStar software (all v.4.1).

### Glycoproteomic data extraction and processing

LaCy Tools data processing package (version 1.0.1, build 9), licensed as free software, was used for rapid automatic peak integration (29). Prior to quantifying the glycoproteomic data, original data files were converted to the open data format mzXML using the ProteoWizard MSConvert tool (version 3.0.19208) (30). Chromatograms were aligned based on the retention times of the five most abundant C3 glycopeptide signals, and targeted peak integration was performed on triply, quadruply, and quintuply charged ion species. Summed spectra were integrated to include at least 90% of the theoretical isotopic pattern, and quality control (QC) parameters (mass accuracy, deviation from the theoretical isotopic pattern, and signal-to-noise ratio) were automatically calculated for each analyte of every sample. Data quality was evaluated by calculating the average QC values for each glycoform and charge state separately, for all the samples analyzed. In order to be included, the criteria were as follows: mass accuracy less than 40 ppm, isotopic pattern quality (IPQ) less than 25%, and signal-to-noise ratio greater than 10. Only the charge states that met all three criteria for each glycoform were summed and quantified. Extracted signals were normalized to total integrated area per glycosylation site to eliminate signal intensity differences between samples.

### Glycoproteomic statistical data analysis

All data was analyzed and visualized using the R programming language (version 3.5.2). Before any statistical modelling, glycoform variables were normalized to total chromatogram area for each glycosylation site. After log-transformation, LC-MS glycopeptide data was batch corrected using ComBat method (R package “sva”) to remove technical variation (31). Plate number of the analyzed samples, which represented the laboratory source of variation, was modelled as a batch covariate. Estimated batch effects were then subtracted from the log-transformed measurements reducing the introduced experimental noise. Assessment of association between C3 N-glycome and various laboratory parameters and clinical status was done using general linear modelling with glycoform area as dependent variable and different biochemical and clinical features as independent variables. To remove the effect of age, sex and duration of the disease, these variables were included as additional covariates. Linear regression model used to test for relationship between C3 N-glycans and T1D duration had only age and sex as additional covariates. The results are presented in odds ratio (for easy comparison of effects’ strength in different groups of categorical variables), or in coefficient which represents the change of normalized glycoform area on a log scale (for continuous data), for each glycoform measured. Considering multiple tests performed, false discovery rate (FDR) was controlled by the Benjamini-Hochberg method with p-value <0.05 considered significant (32).

N-glycome of complement component C3 was then assessed for its ability to differentiate individuals with and without different type 1 diabetes complications. For retinopathy and albuminuria, moderate and severe complications (categories 2 and 3) were grouped together in order to have a binary response: with/without complication. Association of T1D complication status and C3 N-glycome was estimated on all subjects using logistic elastic net regression (α=1, λ=0.02), by comparing area under curve (AUC) of receiver operating characteristic (ROC) curves (R packages glmnet (33) and pROC (34)) obtained from several predictive models. Null model had only sex and age as predictors, while the rest of the models had additional predictors: HbA1c, T1D duration or glycans (full model having them all). To avoid overfitting, 5-fold cross validation was used. Estimated AUCs of ROC curves were compared using bootstrapping (2000 replicates).

## Results

### C3 N-glycome in T1D complications

**Figure 1:**
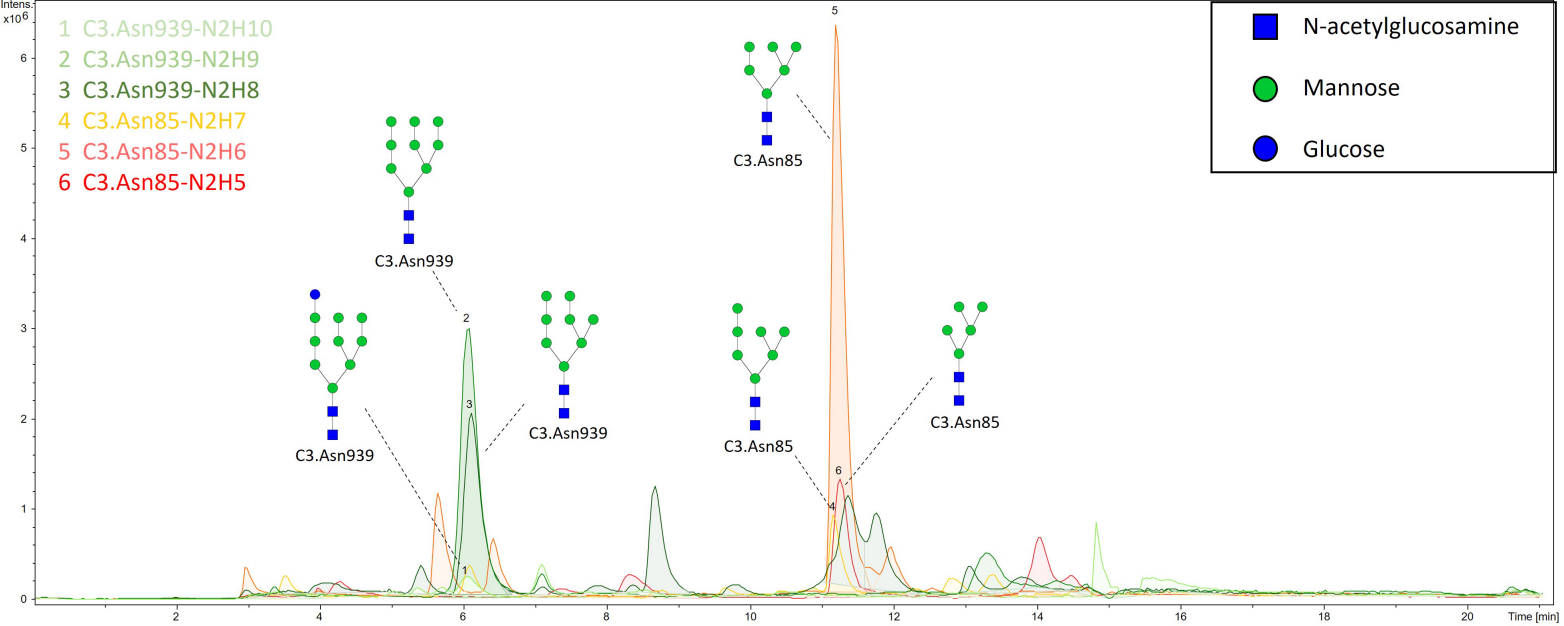
Representative ion chromatogram with extracted traces of all six C3 glycoforms, obtained by our nano-LC-MS system.

N-glycan profiles of complement component C3 have been profiled in 189 T1D subjects with different status of complication severity. Basic characteristics of the study population are given in **Table 1**. The analysis was conducted by high-mannose glycoprotein enrichment from human blood serum using Con A lectin matrix, Glu-C digestion, glycopeptide purification and subsequent nano-LC-ESI-MS technique. Separation was performed based on the peptide backbone of C3 glycopeptides using C18 column thus producing two clusters, originating from two C3 N-glycosylation sites. Each glycoform was normalized to total area per glycosylation site in order to remove variation in signal intensity between samples, allowing for their comparison. A typical chromatogram of human C3 glycopeptides with extracted ion traces forming two clusters, and a total of six glycoforms, is depicted in **Figure 1**. The glycan composition of each site and the abbreviations used for naming the C3 glycoforms are presented in **Table 2**. Complete results of C3 N-glycosylation analysis together with clinical characteristics of the study participants are presented in **Supplementary Table S1**. As we intended to investigate the effect of pathophysiological background of complications themselves, we adjusted our statistical analyses for the effects of age, sex and T1D duration on C3 N-glycome, including them as covariates. As can be seen in **Supplementary Table 1**, only 34 subjects (18%) display multiple complications (considered they have both albuminuria and retinopathy at category 2 or 3). This introduces only slight possibility of one particular complication masking the changes of another, keeping the observed changes truthful.

**Table 1:** Clinical characteristics of the study participants.

Samples (N)	189
Sex (N)	
Male	82 (43%)
Female	107 (57%)
Age (years)	46 (33-56)
T1D duration (years)	14 (7-23)
HbA1c (%)	7.5 (6.6-8.3)
Albuminuria (N)	
1	25 (14%)
2	154 (82%)
3	8 (4%)
Retinopathy (N)	
1	150 (79%)
2	28 (15%)
3	11 (6%)
Smoking (N)	
Non-smokers	131 (70%)
Smokers	57 (30%)
Hypertension (N)	
No	137 (72%)
Yes	52 (28%)
eGFR(mL/min/1.73 m2)	98 (89-111)
Fasting glucose (mmol/L)	7.7 (5.7-9.6)
Post-prandial glucose (mmol/L)	11.55 (8.40 - 14.78)

Data is presented as median (interquartile range) for continuous variables and N (%) for categorical variables.

**Table 2:** Glu-C digested peptide sequence of each N-glycosylation site, its glycan composition and glycoform abbreviation.

N-glycosylation site	Peptide sequence	Glycan composition	Glycoform abbreviation
Asn 85	KTVLTPATNHMGN*VTFTIPANRE	Man_5_GlcNAc_2_	C3.Asn85-N2H5
		Man_6_GlcNAc_2_	C3.Asn85-N2H6
		Man_7_GlcNAc_2_	C3.Asn85-N2H7
Asn 939	GIRMN*KTVAVRTLDPE	Man_8_GlcNAc_2_	C3.Asn939-N2H8
		Man_9_GlcNAc_2_	C3.Asn939-N2H9
		Glc_1_Man_9_GlcNAc_2_	C3.Asn939-N2H10

N-linked asparagine is marked with *. C3.Asn85 – first glycosylation site, C3.Asn939 – second glycosylation site, N – N-Acetylglucosamine, H – hexose.

**Figure 2:**
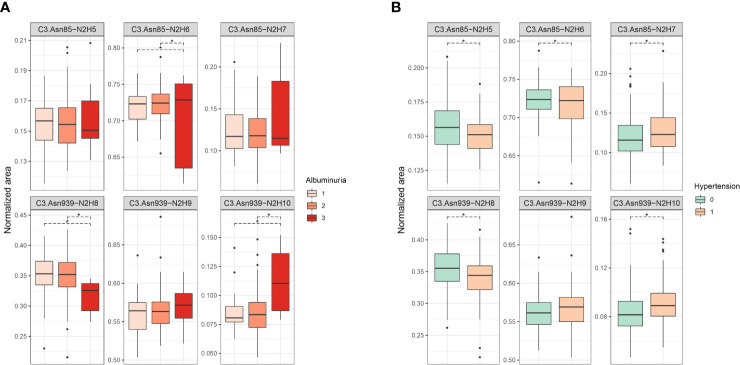
Box plots of batch corrected data showing changes of proportion of glycan structures on both C3 N-glycosylation sites for **(A)** albuminuria, and **(B)** hypertension. Asterisks indicate significant effects (p < 0.05). Dots are outliers. Comparison was done using general linear modelling. C3.Asn85, first glycosylation site; C3.Asn939, second glycosylation site; N, *N*-Acetylglucosamine; H, hexose.

Firstly, we tested the effect of type 1 diabetes complications on C3 N-glycosylation. Hence, we compared measured C3 N-glycome profiles between different stages of T1D complications (albuminuria and retinopathy divided into three categories). For albuminuria, we found that three C3 glycoforms differ significantly between the groups: C3.Asn85-N2H6, C3.Asn939-N2H8 and C3.Asn939-N2H10 (**Supplementary Table S2**). Differences in glycoform abundances in albuminuria are shown as box plots in **Figure 2A**. It can be seen that glycosylation changes are present only in severe albuminuria, while the N-glycan profile of moderate complication is not different from the baseline. The biggest change is the increase in the proportion of the monoglucosylated glycan from glycosylation site C3.Asn939 (OR = 1.344, *p* = 0.002) in category 3 compared to category 2. For retinopathy, glycan changes proved to be smaller and without a consistent trend (**Supplementary Table S3**, **Supplementary Figure S1**). Only one glycoform, C3.Asn939-N2H10, significantly changed. Interestingly, this structure showed the same trend as in albuminuria: an increase in proportion in category 2 compared to category 1 (OR = 1.150, *p* = 0.038).

### C3 N-glycan changes induced by hypertension and smoking

Considered as major risk factors for T1D complication development, we examined effects of hypertension and smoking on relative abundance of different C3 glycopeptides. We divided our subjects in binary groups: smokers/non-smokers, and hypertensive/non-hypertensive. Smoking status in T1D was shown to have no effect on C3 N-glycome, as we have found no statistical differences between in N-glycan profiles between the smokers and non-smokers (**Supplementary Table S4**, **Supplementary Figure S2**). On the other hand, hypertension status significantly affected relative abundance of all except one glycoform (**Figure 2B**, **Supplementary Table S5**). Increase in proportion of glycopeptides with more mannose units is evident on both glycosylation sites. On the C3.Asn85 site, relative abundance of C3.Asn85-N2H7 significantly increased (OR = 1.186, *p* = 2.23×10^-4^), while levels of C3.Asn85-N2H5 and C3.Asn85-N2H6 decreased. On the C3.Asn939 site, a significant increase in the proportion of C3.Asn939-N2H10 (OR = 1.162, *p* = 2.71×10^-4^) was observed, while the level of C3.Asn939-N2H8 dropped and that of C3.Asn939-N2H9 remained unchanged.

### C3 N-glycome association with glycaemic control, eGFR and T1D duration

**Figure 3:**
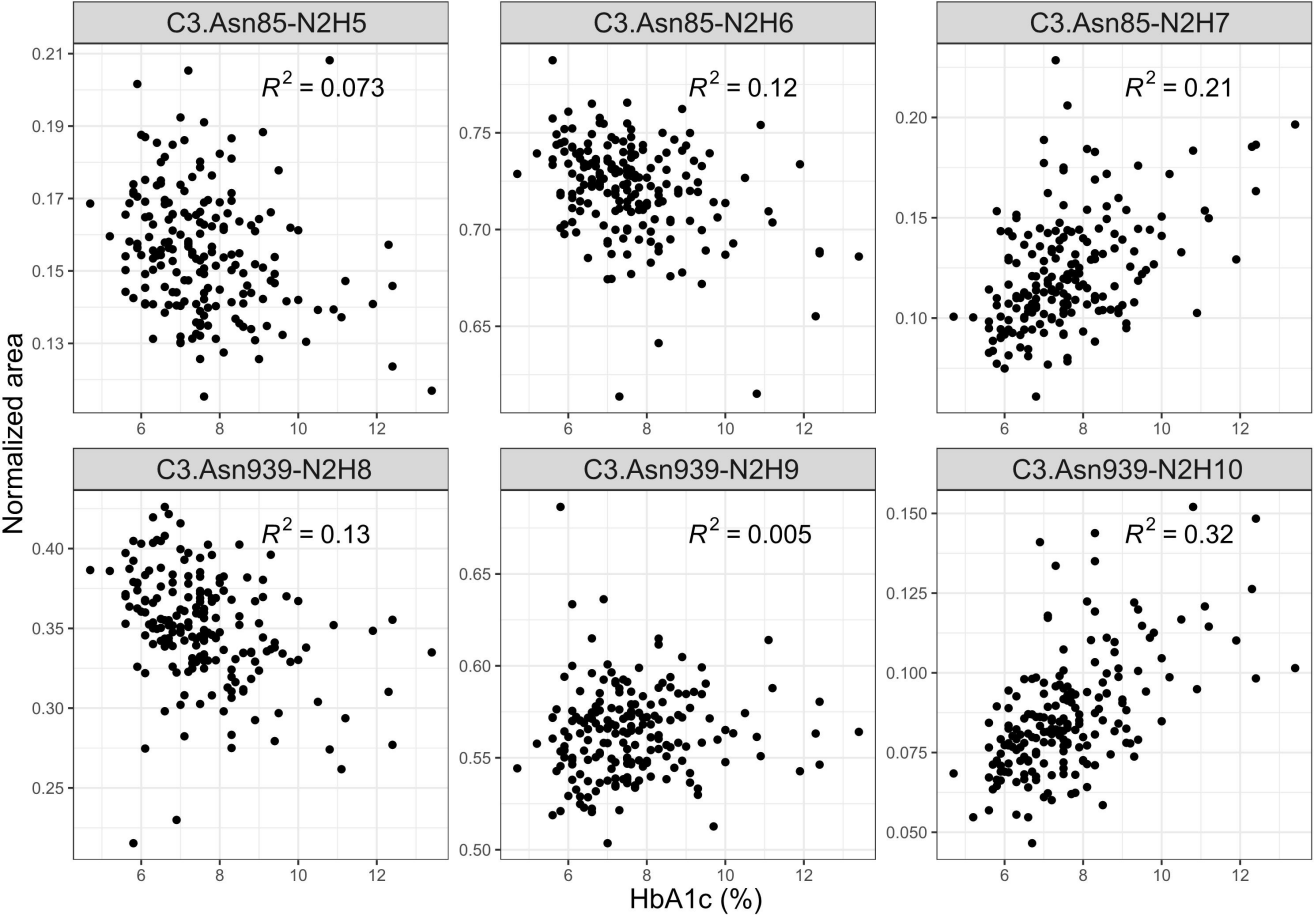
Interdependence of glycaemic control (measured by the level of nonenzymatic glycation of haemoglobin, HbA1c) and normalized areas of C3 glycopeptides.

We also investigated C3 N-glycome associations with fasting and post-prandial glucose levels, as well as with HbA1c, estimated glomerular filtration rate and T1D duration. We found that post-prandial glucose levels are not connected to C3 N-glycans, and that fasting glucose was linked to only C3.Asn939-N2H10 glycoform (*p* = 0.032), as shown in **Supplementary Table S6** and **S7**. eGFR also showed no statistical association with the C3 N-glycome (**Supplementary Table S8**). On the other hand, tested relation of HbA1c levels with the relative abundance of each glycoform proved to be statistically significant. Higher HbA1c was associated with an increase in proportion of glycopeptides with more mannose units on both glycosylation sites (**Figure 3**, **Supplementary Table S9**). The biggest correlation was observed for glycoforms C3.Asn85-N2H7 (*p* = 2.670×10^-7^) and C3.Asn939-N2H10 (*p* = 1.968×10^-17^), both of which are the structures with highest number of mannose units on the glycosylation site. Lastly, type 1 diabetes duration demonstrated no influence on the C3 N-glycome (**Supplementary Table S10**).

### C3 N-glycans as a tool in assessing T1D complication severity

Sequentially, we built a glycan-based discriminative model using logistic elastic net regression for retinopathy, albuminuria and hypertension and compared it to other models to test whether C3 N-glycans are able to distinguish T1D patients with and without complications, and hypertension on top of that. Moreover, we wanted to see if N-glycans as predictors provide additional information that is not already captured by the HbA1c and the disease duration. Models were assessed using a receiver operating characteristic curve analysis: “null” model had only sex and age as predictors, while the rest of the models had additional predictors (“full” model having them all).

**Figure 4:**
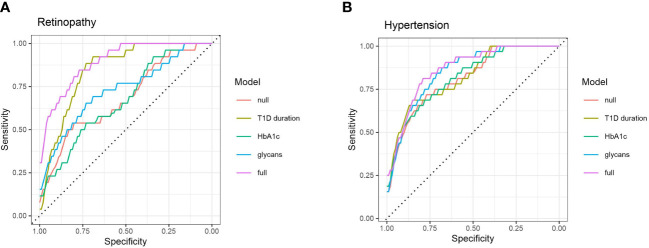
ROC curve analysis of several discriminative models for type 1 diabetes complications: **(A)** retinopathy, and **(B)** hypertension. Null model (red) uses only sex and age as predictors, while full (purple) model includes all of the tested predictors.

For retinopathy, categories 2 and 3 were grouped in order to have a binary response: with/without complication. The models are presented in **Figure 4A**. Although disease duration contains most of the information needed for distinguishing T1D subjects based on their retinopathy status (AUC 0.847, 95% CI 0.764 – 0.913), incorporating glycans further enhances discriminative power (AUC 0.895, 95% CI 0.823– 0.951). Interestingly, glycans on their own (AUC 0.730, 95% CI 0.603 – 0.842) performed better than traditional markers such as HbA1c (AUC 0.673, 95% CI 0.558 – 0.782). For albuminuria, C3 N-glycans showed no discriminative power for distinguishing T1D subjects with albuminuria from subjects without this complication (**Supplementary Figure S3**).

Discriminative models of hypertension are presented in **Figure 4B**. HbA1c (AUC 0.817, 95% CI 0.723 – 0.892) and T1D duration (AUC 0.820, 95% CI 0.729 – 0.899) are not much more informative predictors than the null model (AUC 0.812, 95% CI 0.719 – 0.889), while the glycans have somewhat more solid discriminative power (AUC 0.852, 95% CI 0.769 – 0.920), close to the full model (AUC 0.864, 95% CI 0.784 – 0.925).

## Discussion

Changes of C3 N-glycome in young type 1 diabetes population has already been reported, with T1D subjects having significant increase in the proportion of the unprocessed glycan structures, i.e. structures with more mannose units, on both of the glycosylation sites (10). Bearing in mind that the population studied consisted of early onset T1D children and adolescents, we wanted to investigate whether C3 N-glycome could also possibly reflect complications accompanying the disease over the years. To the best of our knowledge, this is the first study to address individual variation of complement component C3 N-glycosylation profiles in type 1 diabetes complications, despite the strong basis for expecting such alterations to occur in these conditions (11, 12, 21, 25, 26). C3 protein glycosylation exhibited several changes in T1D complications, as well as in hypertension accompanying this condition. We observed that subjects with severe albuminuria showed an increase in proportion of unprocessed glycan structures, on the C3.Asn939 site. Same trend towards glycoforms with more mannose units was also seen in T1D hypertension, but this time for both of the glycosylation sites. Additionally, the most unprocessed C3 glycoform, C3.Asn939-N2H10, significantly increased in non-proliferative retinopathy and across albuminuria stages. Interestingly, changes in both T1D severe albuminuria and hypertension correspond to alterations observed in young T1D subjects compared to healthy control determined in our previous study (10), as both type 1 diabetes itself and its accompanying complications shift the C3 N-glycan profile in the direction of unprocessed glycans with more mannose units.

Previous studies have already demonstrated interdependence of high mannose serum N-glycans and eGFR in type 1 diabetes subjects (11, 12). These studies found a shift toward more complex total serum N-glycans with a progressive renal decline (as measured by steeper decline in eGFR). Although among observed associations were high-mannose glycans, C3 seems not to be a cause of this changes as we revealed that C3 N-glycome is not associated with eGFR values. It should be noted, however, that our study included subjects with normal/high or mildly decreased renal function, assessed as eGFR >60 ml/min/1.73 m^2^. On the other hand, distinct total plasma N-glycome changes in type 2 diabetes subjects with prevalent retinopathy have been reported, with strongest association being the ratio of high-mannose to hybrid glycans (35). Since complement component C3 is a major source of highly mannosylated glycans in plasma N-glycome, the observed change of this type of glycans could be a consequence of different C3 glycosylation pattern, as we herein report that C3 N-glycome is sensitive to T1D non-proliferative diabetic retinopathy. Regarding hypertension, it is known that both systolic and diastolic blood pressure are associated with total plasma N-glycome (36), and that a specific protein like IgG notably contributes to pathogenesis of hypertension (15, 37). This study showed significant changes of C3 N-glycans profile in T1D hypertension, suggesting distinct role of this protein in its development. Nevertheless, as some of the subjects in our study were on antihypertensive treatment, it should be noted that observed C3 N-glycome changes could be affected by the medications, with effect of hypertension itself being obscured. Regarding HbA1c, poor glycaemic control in both type 1 and type 2 diabetes has been shown to alter N-glycome, with elevated HbA1c levels associated with increased complexity of total plasma glycan structures (11, 38). Even so, these studies could not pinpoint the exact protein that becomes affected. Here we demonstrated that poorer glycaemic control in type 1 diabetes changes glycosylation even on the level of a single protein.

Prolonged hyperglycaemia typical for type 1 diabetes causes glucose-induced tissue damage, which is hypothesized to be mediated through a range of pathways, including increased flux of glucose through the hexosamine biosynthetic pathway (HBP). Under conditions of hyperglycaemia the percentage of total glucose utilized through the HBP could be enhanced, leading to increased generation of the end product uridine diphosphate N-acetyl-glucosamine (UDP-GlcNAc), a substrate used for protein glycosylation (6, 39). This was proposed as one of the mechanisms for the observed plasma N-glycosylation changes in diabetes, such as the increase in proportion of highly branched N-glycans (11, 38). However, this explanation is less likely for C3, as it only contains high-mannose glycans which are not primarily dependent on the UDP-GlcNAc. The elevation in proportion of unprocessed high-mannose glycans suggests a premature termination of the glycosylation pathway and a problem during the synthesis that prohibits deletion of present sugar residues (8). This could possibly be explained by endoplasmic reticulum (ER) stress typical for renal and pancreatic cells in type 1 diabetes (40, 41), and the fact that kidneys and pancreatic islets also highly express C3, contributing to its total production with about 4% each (42, 43). Complement component C3 N-glycome changes are maybe coming from this local C3 aberrant glycan profile.

Total plasma N-glycans have already been shown to improve type 2 diabetes and cardiovascular diseases prediction beyond established risk markers (44). In this study we suggest that C3 N-glycans could be a new tool for assessment of glycaemic control, as they were found to be associated with commonly used diagnostic markers of T1D, such as HbA1c and fasting glucose levels. This is further backed up by the fact that some aspects of poor glycaemic control relevant to complications development are not captured by these traditional biomarkers, as seen in **Figures 4A, B**. Discriminative models of T1D complications with HbA1c as additional predictor showed similar distinguishing power as null models, while glycans seemed to possess a better discriminative power. All these findings suggest that C3 N-glycans may capture an interconnection of the glycaemic control with T1D complications that is not already captured by the HbA1c.

As observed C3 N-glycome alterations proved to be independent of the disease duration, and reaffirmed the changes seen in early onset T1D compared to the healthy controls, they could potentially be utilized as a T1D complication biomarker. Namely, these glycan changes may be present at the time of the T1D diagnosis (or even before), and indicate the individuals with higher risk of developing the complications accompanying the disease. Even though the presence of multiple complications in studied subjects makes it hard to link exact C3 N-glycan profile with particular complication, it seems that C3 N-glycome possibly reflects general T1D complication status. Considering the significant increase in the proportion of particular glycoform (C3.Asn939-N2H10) in the patients with incipient albuminuria and mild/moderate retinopathy when compared to complication-free subgroup, it seems that C3 N-glycans could potentially be exploited to detect early complications and/or monitor the disease progression. Nevertheless, as this type of study limits us in conclusions whether observed changes are due to complications, or they precede them, potential value of our C3 N-glycome findings in risk assessment of T1D complications should additionally be studied and confirmed in prospective studies.

The biggest strength of this study is the site-specific N-glycosylation LC-MS analysis of human C3 which guarantees that the glycosylation changes are protein specific and are not confounded by contaminating glycoproteins. Thus, our findings are indeed a consequence of variation in glycosylation of C3, and not just a result of its concentration variation. We also carried out extensive covariate adjustment of the measured data, and performed discriminative modelling using cross-validation and methods to avoid overfitting. One study limitation that we need to acknowledge is the lack of controls, which would make possible to detect N-glycome changes not only in the progression of the complications, but also its distinction from healthy individuals. Besides, due to the cross-sectional design of our study we cannot speculate on causality of the observed glycan changes, nor can we monitor long-term outcomes. Even so, this study provided new insights into C3 glycan changes occurring in T1D complications and strengthened the relevance of C3 N-glycosylation research in diabetes.

## Data availability statement

The original contributions presented in the study are included in the article/**Supplementary Material**. Further inquiries can be directed to the corresponding author.

## Ethics statement

The studies involving human participants were reviewed and approved by School of Medicine Ethics Committee, University of Zagreb. The patients/participants provided their written informed consent to participate in this study.

## Author contributions

OG conceived, designed, and supervised the study. DŠ, JŠ, MTi, TK, GL, TB, ML, SR, MTo, MN and LS-D contributed to the data collection, acquisition or analysis and interpretation of data. DŠ and OG wrote the manuscript. OG is the guarantor of this work and, as such, had full access to all the data in the study and take responsibility for the integrity of the data and the accuracy of the data analysis. All authors contributed to the article and approved the submitted version.

## Supplementary material

Supplementary Figure 1 Box plots of batch corrected data showing changes of proportion of glycan structures on both C3 N-glycosylation sites for retinopathy. Asterisks indicate significant effects (p < 0.05). Dots are outliers. Comparison was done using general linear modelling. C3.Asn85 – first glycosylation site, C3.Asn939 – second glycosylation site, N – *N*-Acetylglucosamine, H – hexose.

Supplementary Figure 2 Box plots of batch corrected data showing no changes of proportion of glycan structures on both C3 N-glycosylation sites for smoking status. Dots are outliers. C3.Asn85 – first glycosylation site, C3.Asn939 – second glycosylation site, N – *N*-Acetylglucosamine, H – hexose.

Supplementary Figure 3 ROC curve analysis of several discriminative models for type 1 diabetes albuminuria. Null model (red) uses only sex and age as predictors, while full (purple) model includes all of the tested predictors.
