# Supplementary figures and images for: Human complement component C3 N-glycome changes in type 1 diabetes complications
Source: Front Endocrinol (Lausanne). 2023 May 24;14:1101154. doi: 10.3389/fendo.2023.1101154 (PMC10244649; doi:10.3389/fendo.2023.1101154)

Normalized area

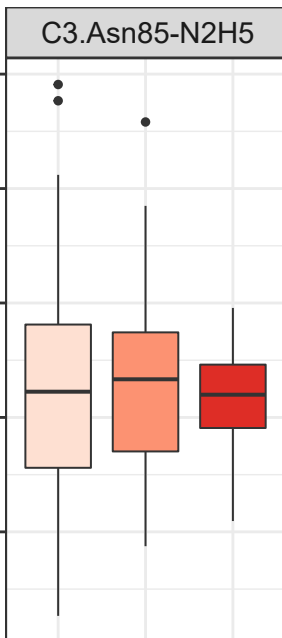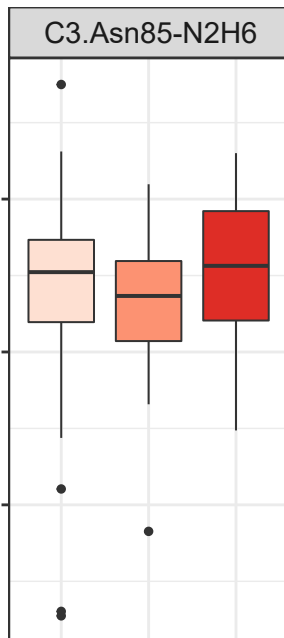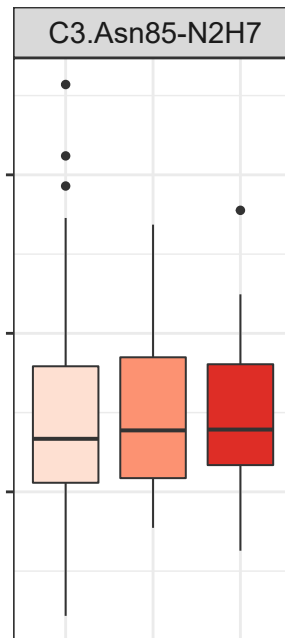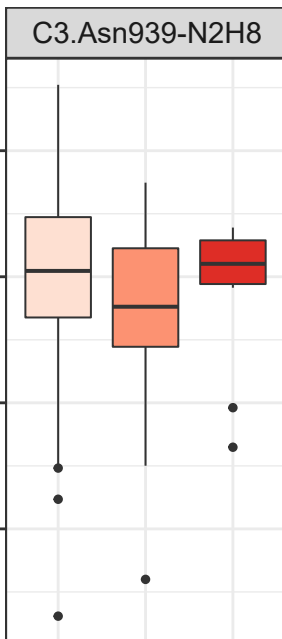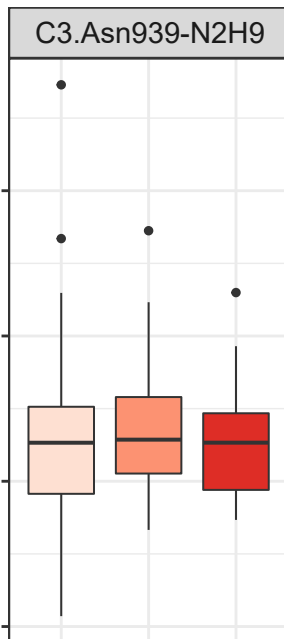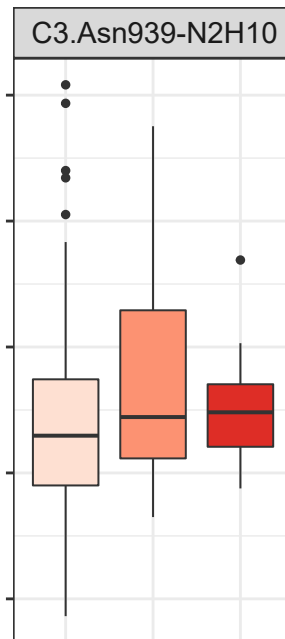

Retinopathy

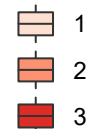

Supplement: Supplementary Figure 1 — Box plots of batch corrected data showing changes of proportion of glycan structures on both C3 N-glycosylation sites for retinopathy. Asterisks indicate significant effects (p < 0.05). Dots are outliers. Comparison was done using general linear modelling. C3.Asn85 – first glycosylation site, C3.Asn939 – second glycosylation site, N – N-Acetylglucosamine, H – hexose. [file Image_1.pdf]

Normalized area

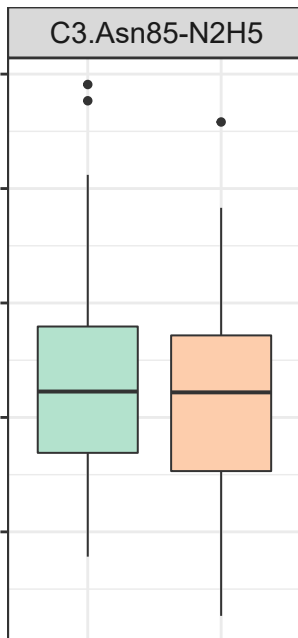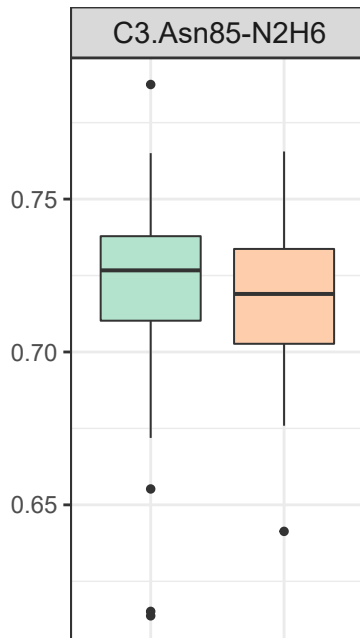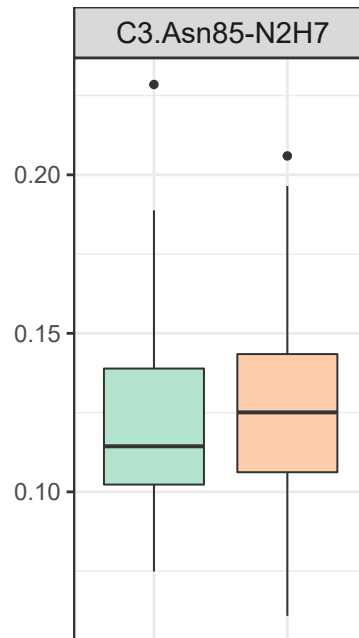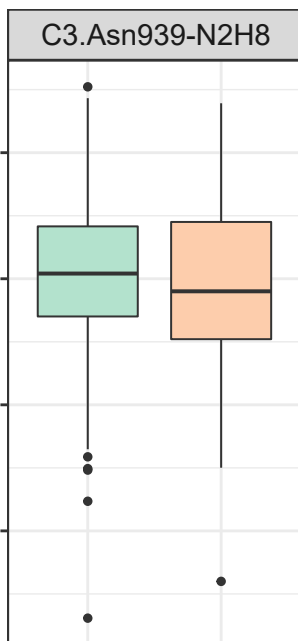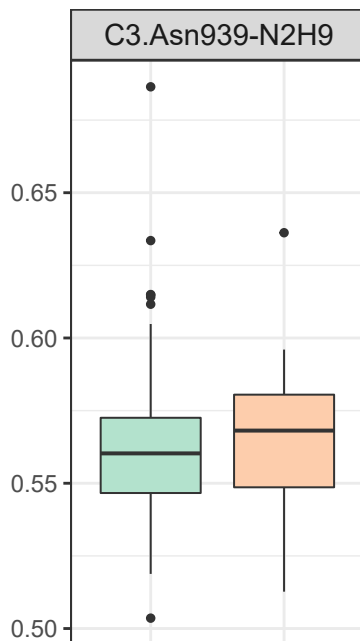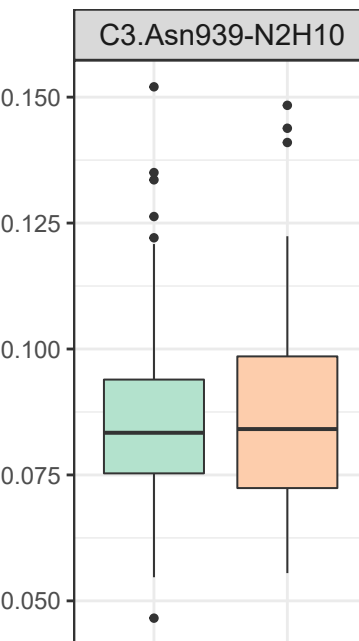

Smoking

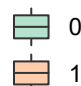

Supplement: Supplementary Figure 2 — Box plots of batch corrected data showing no changes of proportion of glycan structures on both C3 N-glycosylation sites for smoking status. Dots are outliers. C3.Asn85 – first glycosylation site, C3.Asn939 – second glycosylation site, N – N-Acetylglucosamine, H – hexose. [file Image_2.pdf]

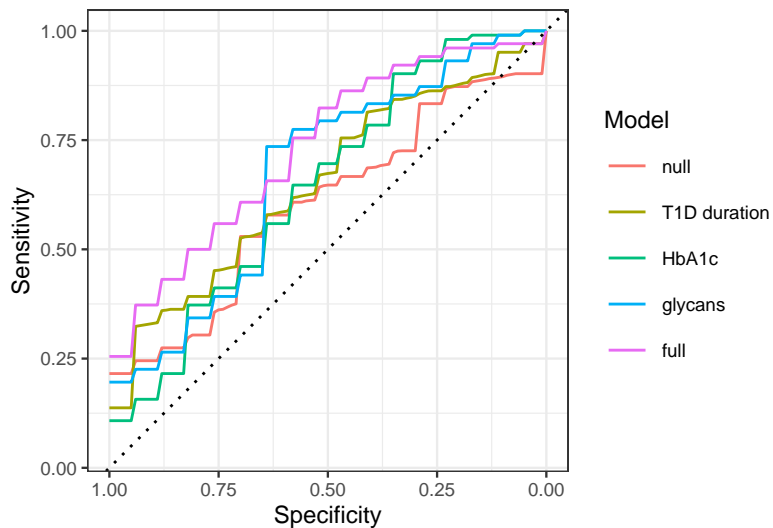

Supplement: Supplementary Figure 3 — ROC curve analysis of several discriminative models for type 1 diabetes albuminuria. Null model (red) uses only sex and age as predictors, while full (purple) model includes all of the tested predictors. [file Image_3.pdf]
